# Kinematic and dynamic analysis of walking dynamic balance stability in children with spastic cerebral palsy diplegia

**DOI:** 10.3389/fbioe.2025.1604658

**Published:** 2025-10-24

**Authors:** Tingting Ma, Qi Zhang, Tiantian Zhou, Hongbo Zhao, Yan He, Tianyang Feng, Qing Yue, Xiaosong Li, Yanqing Zhang

**Affiliations:** ^1^ Department of Physical Therapy for Children, Beijing Boai Hospital, China Rehabilitation Research Center, Beijing, China; ^2^ Rehabilitation Medical School of Capital Medical University, Beijing, China

**Keywords:** cerebral palsy, spastic cerebral palsy, exercise intervention, joint anglechanges, temporal and spatial gait parameters, kinematics, statistical parametricmapping

## Abstract

**Objective:**

This study aims to compare biomechanical features during walking between children with spastic cerebral palsy (SCP) and typically developing children, providing evidence to improve walking ability and prevent falls in children with SCP.

**Methods:**

The study included 28 children with SCP from the paediatric physiotherapy department of the China Rehabilitation Research Centre (March 2023 to September 2024) and 28 typically developing children from a Beijing primary school as controls. Participants wore tight-fitting clothing to ensure clear visibility of reflective markers. A Vicon 3D motion capture system and AMTI force plates were used to collect data on temporal-spatial parameters, dynamic balance and kinematic parameters during gait cycles. Measurements included walking speed; step frequency, width and length; single-foot support time; peak displacements of the centre of mass (COM) and the centre of pressure; and joint angles of the pelvis, hip, knee and ankle in multiple planes.

**Results:**

Children with SCP showed significantly lower values in walking speed, stride length, step length and single-foot support time than the controls (P < 0.05). Conversely, cadence, stride time and double support time were higher in children with SCP than in the controls. Children with SCP showed greater peak COM displacement in the coronal plane but lower in the sagittal plane than the controls (P < 0.05). Significant differences were found in the range of motion of left lower extremity joints across various phases of the gait cycle (P < 0.05).

**Conclusion:**

Children with SCP exhibit distinct gait patterns and dynamic balance challenges compared with their typically developing peers, underscoring the importance of personalised rehabilitation treatments to enhance their walking abilities and prevent falls.

**Clinical Trial Number:**

ChiCTR2300071226.

## 1 Introduction

Cerebral palsy (CP) is a group of persistent syndromes that affect motor and postural development as well as activity limitations (Chinese association of rehabilitation medicine pediatric rehabilitation committee, 2022). Among these, spastic CP (SCP) is characterised by impaired movement and postural control and poor walking balance due to increased muscle tone and the persistence of primitive reflexes (Wu et al., 2017). In clinical practice, a variety of balance scales and static balance test systems are frequently used to evaluate the balance abilities of children with SCP. However, the outcomes are largely subjective and do not capture the assessment of children’s dynamic balance capabilities (Damiano et al., 2017). The ability to maintain dynamic balance is a fundamental prerequisite for self-care in children with SCP (Pourazar et al., 2023). Conversely, for children with SCP who retain some walking ability, good dynamic balance provides the foundation for fall prevention (Pourazar et al., 2019). Accordingly, enhancing dynamic balance has become a key objective of physical therapy (PT) interventions for SCP.

The forward movement of a person walking on level ground exhibits a “pendulum pattern,” characterised by two key variables: the centre of mass (COM) and the centre of pressure (COP). The COM represents the average location of the body’s mass distribution, typically near the pelvis in an upright stance, but it shifts dynamically during movement. It is a key parameter for assessing balance and gait stability. The COP is defined as the point of application of the ground reaction force on the supporting surface during standing or walking. It reflects the dynamic interaction between body mass distribution and external forces, serving as an important indicator of postural stability and gait control during movement (Wallard et al., 2018). Both are frequently employed metrics for the assessment of dynamic balance function in children with SCP and typically developing children (Kimoto et al., 2021). As highlighted by Bruijn et al., children with CP exhibit increased gait variability and reduced dynamic stability margins compared with their typically developing peers. These findings suggest that balance control during movement is considerably impaired in this population, potentially increasing the risk of falls and limiting functional independence (Bruijn et al., 2013). Therefore, studying the kinematics and dynamics of the COM and the COP during walking could provide valuable insights into the underlying mechanisms of gait instability in children with SCP.

The Vicon 3D Motion Capture System provides an advanced, quantitative tool for objectively evaluating gait characteristics and kinematic and kinetic parameters. It is especially valuable for paediatric studies, where research is still emerging (Favetta et al., 2022; Tsitlakidis et al., 2022). Compared with the existing body of research on 3D gait in adults, the field of paediatric motor function studies remains in its infancy. Although adult gait studies are abundant, studies on paediatric motor function are scarce, particularly those comparing children with CP to their typically developing peers. Moreover, most existing studies have focused on changes in temporal and spatial gait parameters, with even fewer exploring differences in dynamic balance during walking (Wallard et al., 2017; Mazzarella et al., 2020). Understanding the relationship between locomotion and force is essential for guiding PT in children with CP (Tinker et al., 2022). Additionally, examining changes in joint angles throughout the gait cycle is important, as previous studies have mainly analysed peak joint angle activity, not fully reflecting overall joint changes (Opara et al., 2017). In contrast, one-dimensional statistical parametric mapping (SPM) can assess waveform data based on the temporal characteristics of joint angles, offering high statistical value (Yona et al., 2024; Dobler et al., 2023; Luciano et al., 2021). Therefore, this study will use the Vicon 3D motion capture system combined with the SPM method to evaluate the dynamics and kinematics of ground walking in children with SCP and typically developing children. Specifically, differences in COM, COP and lower limb joint angles in the sagittal, coronal and transverse planes during ground walking are compared. The aim is to explore the dynamic and kinematic differences between children with SCP and their typically developing peers, providing reliable quantitative data for restorative gait training and theoretical support for fall prevention strategies.

## 2 Materials and methods

### 2.1 General information

Between March 2023 and September 2024, 28 children with SCP who received conventional PT at the Department of Paediatric Physical Therapy of the China Rehabilitation Research Centre were recruited as the experimental group. Additionally, 28 typically developing children of the same age were randomly selected from Majiapu Primary School in Feng Tai District, Beijing, to serve as the control group.

The inclusion criteria:

Experimental group: 1) aged 6–12 years old; 2) diagnosis confirmed by a paediatric neurologist or rehabilitation physician according to the criteria for spastic diplegia in the 2022 edition of the *Chinese Cerebral Palsy Rehabilitation Guidelines* (Chinese association of rehabilitation medicine pediatric rehabilitation committee, 2022); 3) Gross Motor Function Classification System levels I–III; 4) able to complete a 10-m walk test independently without the aid of any assistive devices.

Control group: 1) aged 6–12 years old; 2) no disorders that obviously affect physical ability; 3) no obvious abnormalities in either lower limb, such as foot deformities, genu varum or genu valgum.

The exclusion criteria: 1) participants who received botulinum toxin injections within 3 months before the test; 2) received orthopaedic surgery within 6 months before the test; 3) trauma to the lower limbs, unstable fracture, knee pain or limited mobility; 4) unable to tolerate training, understand test instructions or cooperate; 5) Modified Ashworth Scale score of at least 3; 6) obvious isometric shrinkage in both lower limbs; 7) receipt of anticonvulsant medications (e.g., baclofen, benzodiazepines) within 6 months before testing; 8) joint contractures that substantially affect gait patterns, such as ankle dorsiflexion less than 10°.

Exclusions and withdrawals: 1) automatic termination of the trial; 2) violation of the trial protocol; 3) termination of the trial due to an adverse event, provided that such events were accounted for in the assessment of adverse reactions. The study was approved by the Ethics Committee of the China Rehabilitation Research Centre (No. 2022-043-2). Written informed consent was obtained from all legal guardians. In addition, age-appropriate consent was obtained from children with sufficient cognitive capacity to understand the purpose and procedures of the study.

### 2.2 Study methods

**FIGURE 1:**
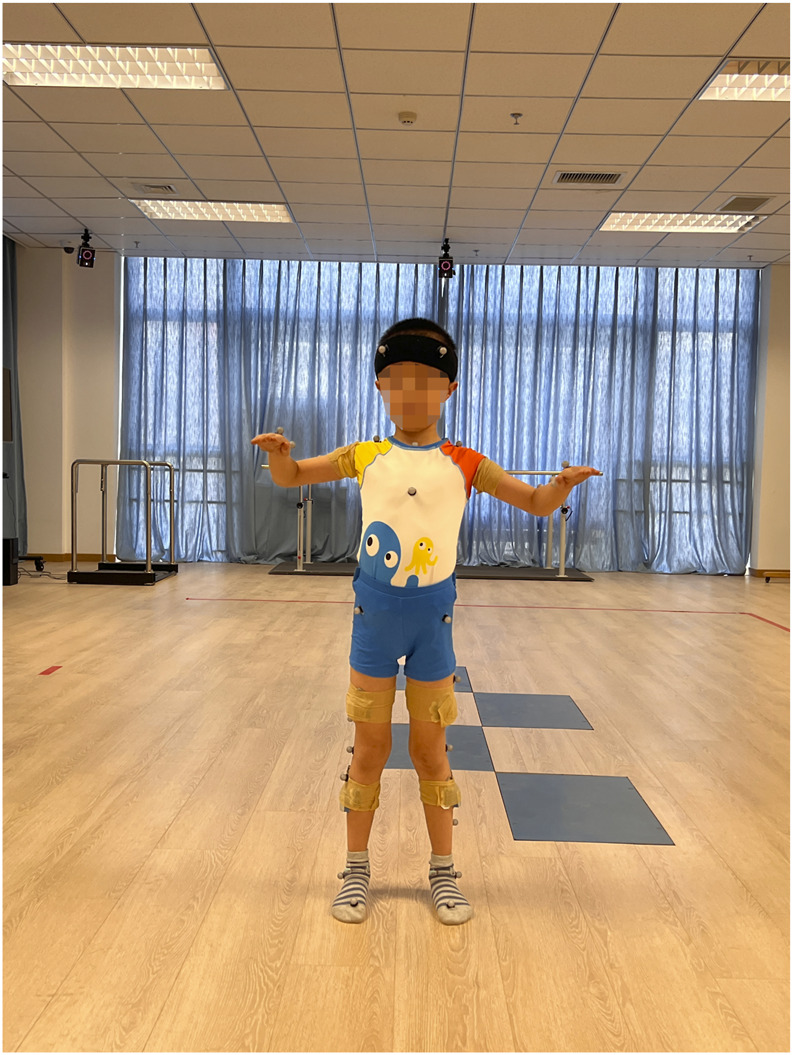
Marking points (front view). From top to bottom: Lateral forehead (temples) bilaterally, shoulder crests bilaterally, upper arms, lateral elbow joint line, forearms, medial and lateral wrist joint line, 2nd metacarpophalangeal joints, clavicular fossa, sternal styloid, anterior superior iliac spines bilaterally, midpoint of lateral thighs of both lower extremities, knee joint line, midpoint of lateral calves, lateral ankles, 1st phalangeal joints.

**FIGURE 2:**
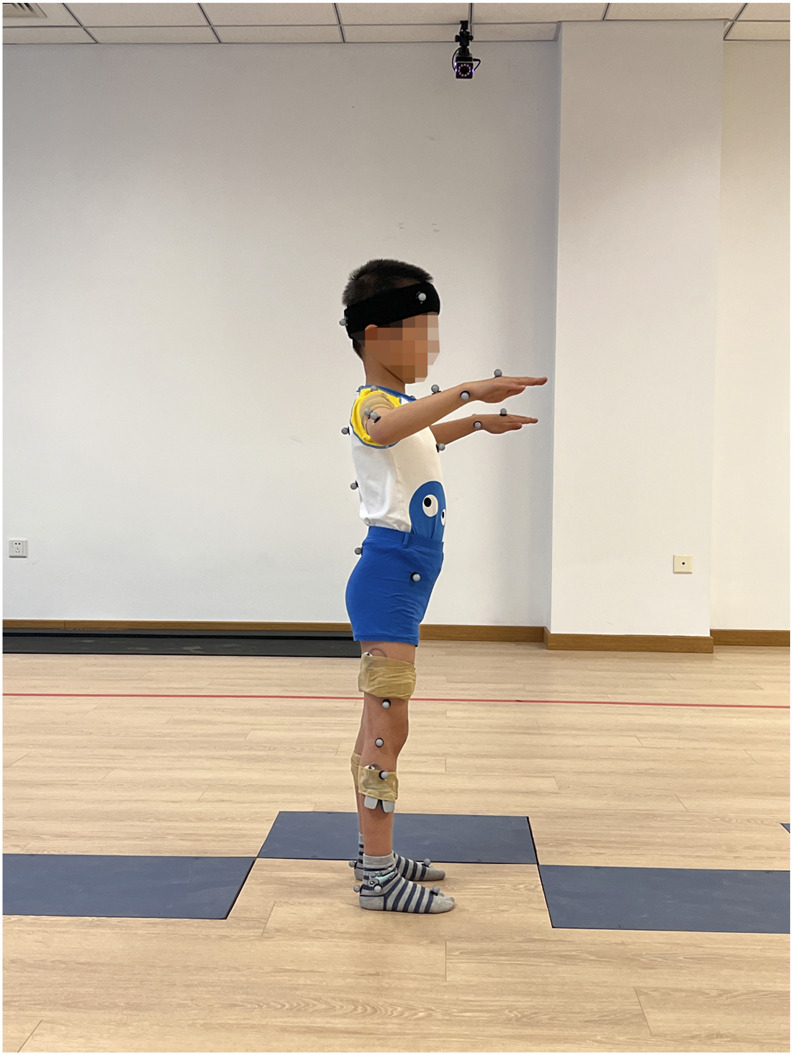
Marking points, (side view, right side). From top to bottom: Lateral right forehead (solar plexus), right posterior occiput, C7 and T10 body surface landmarks, right mid-scapula, right acromion, upper arm, lateral elbow joint line, forearm, medial and lateral wrist joint line and 2nd metacarpophalangeal joint, clavicular fossa and sternal pedicle, right anterior supra-iliac spine, right posterior supra-iliac spine, right lower extremity lateral thigh mid-point, knee joint line, lateral calf mid-point, lateral ankle, heel, and 1st phalangeal joint.

**FIGURE 3:**
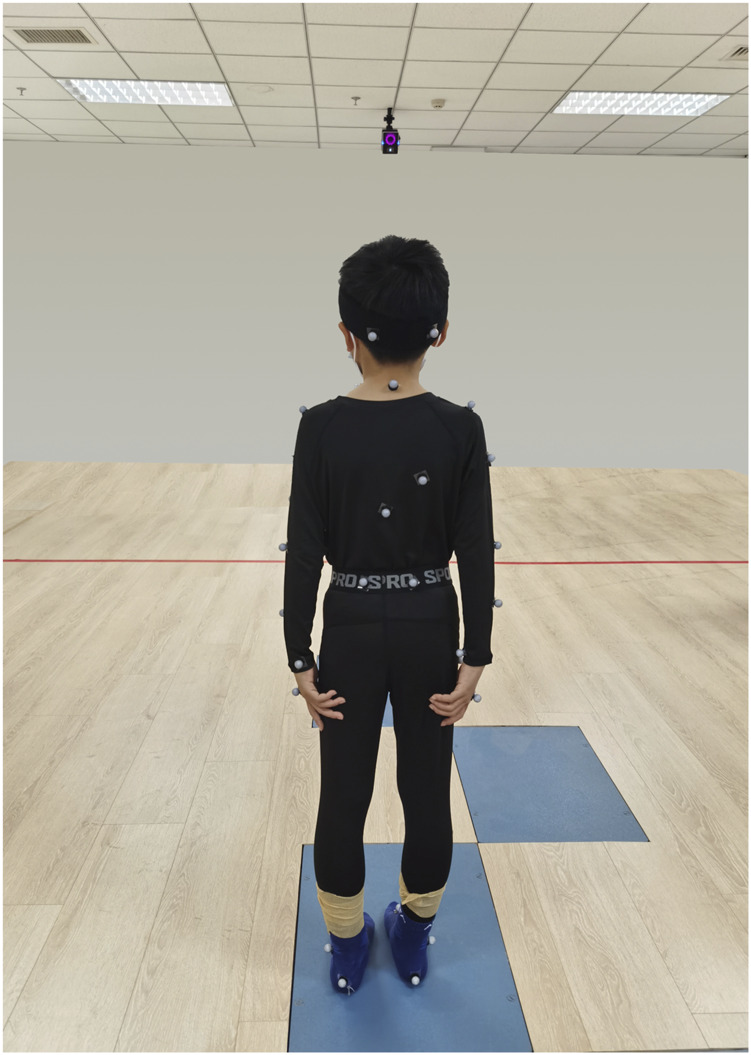
Marking points (back view). From top to bottom: Bilateral posterior occiput, C7 and T10 body surface landmarks, right mid-scapula, bilateral acromion, upper arm, lateral elbow joint line, forearm, lateral wrist joint line and 2nd metacarpophalangeal joints, bilateral posterior superior iliac spines, bilateral lower extremity lateral thigh midpoints, knee joint line, lateral calf midpoints, lateral ankles, heels.

The 28 children with SCP and 28 typically developing children were required to wear tight-fitting tops, shorts and short socks to ensure clear visibility of reflective markers and prevent clothing from obscuring them. For children with CP, there may be some degree of asymmetry or deformity in the ankle joint structure, so clinical anatomical landmarks (such as the medial malleolus, lateral malleolus and tibial tuberosity) were used as reference points to improve the stability and accuracy of data collection. The therapist measured the participant’s height, weight, left and right lower limb length (distance from the anterior superior iliac spine to the medial ankle), left and right knee width (distance between the medial and lateral sides of the knee), left and right ankle width (distance between the inner and outer ankles), left and right elbow width (distance between the medial and lateral sides of the elbow), left and right metacarpal thickness (thickness of the thickest part of the metacarpal bone on the palm), left and right wrist width (distance between the medial and lateral wrists) and left and right shoulder thickness (distance from the peak of the shoulder to the centre of shoulder joint motion). After entering the anthropometric data into the tester’s computerised system, 39 reflective markers were affixed to the participant’s body according to the specifications of the Plug-in Gait Full Body acquisition software package. From superior to inferior, the following anatomical landmarks were marked: bilaterally on the lateral forehead (temple) and posterior occipital regions; the C7 and T10 vertebral landmarks; the clavicular fossae and sternal manubrium; the right mid-scapula; the bilateral acromions; the upper arms; the lateral elbow joint lines; the forearms; and the wrist joint lines. Additional markers were placed on the inner and outer second metacarpophalangeal joints; the bilateral anterior and posterior superior iliac spines; the lateral mid-thighs; the knee joint lines; the lateral mid-shanks; the lateral ankles; the heels; and the first toes (Figures 1–3).

A Vicon 3D motion capture system (Vicon, Oxford, UK) was used to capture and process the trajectories and forces at various body surface marker points during walking. The Vicon Nexus system recorded whole-body kinematic parameters. Eight V5 infrared cameras were mounted on the indoor walls for motion capture, with an acquisition frequency of 100 Hz, using 39 reflective markers (14 mm diameter). Kinetic data were collected using 4 AMTI force plates (AMTI, BP400600, USA), each with an acquisition frequency of 1,000 Hz. The plates measured 60 × 40 × 8.3 cm, with a maximum vertical force of 8.8 kN and a maximum lateral force of 4.4 kN. A test frequency of 2,000 Hz was employed, and the motion capture and force plate systems were synchronised for data acquisition. Marker and force data were filtered to enhance signal quality. Kinematic data (marker trajectories) were processed using a low-pass Butterworth filter with a cut-off frequency of 6 Hz. Ground reaction force data were similarly filtered using a low-pass Butterworth filter with a 20 Hz cut-off frequency. All filters were applied using zero-lag bidirectional filtering to prevent signal delay and ensure temporal synchronisation. These procedures removed high-frequency noise while preserving the true motion characteristics, resulting in smooth and physiologically accurate curves.

The test was conducted by the same physiotherapist with extensive experience in gait analysis. Before the test began, the camera parameters were calibrated to ensure each camera captured the full field of view and complete range of motion while avoiding any sources of interference in the recording environment or from other cameras. The system was calibrated using a T-shaped calibration rod with known marker spacing. The tester moved the calibration rod throughout the capture area to ensure accurate reconstruction of the three-dimensional space. This process enabled the system to calculate the position and orientation of the cameras, as well as internal parameters (e.g., focal length, lens distortion) (Smirnova et al., 2022). To minimise environmental interference, reflective surfaces in the test area were removed before data acquisition, and all reflective markers were visually confirmed.

Before the test, the participants were instructed to walk back and forth outside the lateral force platform several times at their habitual posture and speed. They were asked to keep their head up, look straight ahead, raise their arms forward, abduct the shoulder joints to 90° and flex the elbow joints to 90°. The spatial origin of each marker point was recorded by the system to establish a static model. Subsequently, each participant walked the length of a 10-m walkway over the force platforms at a natural pace, 5 times, in a straight line. Gait data were collected during walking to ensure that both lower limbs contacted different force platforms and that each marker remained within the infrared capture zone, thereby avoiding any factors that might affect the accuracy of the gait analysis. Each limb was selected for a complete gait cycle (from heel strike to toe-off and back to heel strike), manually identified by the researchers based on visual observation and the vertical ground reaction force curve. Test data were excluded if there were sudden changes in speed, steps that missed the force plate, or incomplete gait cycles. Two physiotherapists, blinded to the participants’ information, independently reviewed the selected gait cycles. In cases of disagreement, a third reviewer participated in the discussion to finalise the selection. For each participant, 3–5 gait cycles were recorded for subsequent data processing.

### 2.3 Assessment of indicators

To ensure the rigour of the trial, two blinded physiotherapists were paired to assess all indicators.

#### 2.3.1 Temporal and spatial gait parameters

Walking speed, step frequency, step width, step length, stride length, single-leg support time and double-leg support time were recorded for both groups of children during a complete gait cycle.

#### 2.3.2 Dynamic balance parameters

The peak displacement of the COM and the COP in the coronal and sagittal planes during a complete gait cycle was recorded for both groups. The maximum–minimum method (i.e., extremes in both directions are considered) was used to more fully reflect the range of variation in the equilibrium state during the gait cycle. In addition, the mean ± standard deviation was calculated to assess consistency and variability in stability.

#### 2.3.3 Kinematic parameters

The angles of the pelvic, hip, knee and ankle joints in the coronal, sagittal and horizontal planes were recorded from the children in both groups during one complete gait cycle.

In order to ensure the validity and rationality of the statistics, and to take into account the often asymmetry of limb movement disorder severity in children with spastic cerebral palsy, each participant in the experimental group in this study identified their more severely affected lower limbs through clinical evaluation. Subsequently, kinematic data from the more severely affected side were selected for analysis (Vitrikas et al., 2020). The control group was matched accordingly, and the lower limb data on the same side of the body as the experimental group was selected for analysis.

### 2.4 Statistical analysis

The temporal and spatial gait parameters and dynamic balance parameters were analysed using SPSS 27.0 statistical software. First, the data were subjected to a normality test. Measurements conforming to a normal distribution were expressed as the mean ± standard deviation (x ± s), and comparisons between groups were made using an independent samples *t*-test. Measurements not conforming to a normal distribution were expressed as the median, and comparisons between groups were made using a Mann–Whitney U test. Fisher’s exact test was conducted on the count data. For categorical variables (e.g., gender, gait type), the chi-squared test was used to compare groups. All statistical tests were two-sided, with the significance level set at α = 0.05.

The SPM method was employed to analyse kinematic parameters using two-tailed paired SPM *t*-tests to determine differences in joint angles across the coronal, sagittal and horizontal planes. The angular statistical analysis was performed in Python using the open-source SPM1D software package.

The required sample size was estimated using G*Power software. The independent samples *t*-test was selected as the statistical test, with an effect size of 0.5, a significance level of 0.05 and a power of 80%, resulting in a total of 28 cases per group.

## 3 Results

A total of 56 participants were included in this study, comprising both the experimental and control groups. No participant showed signs of dropout, and all data were deemed valid and included in the subsequent analysis.

### 3.1 Comparison of general information

No significant differences were observed between the two groups in age, gender, height, weight and lower limb length (P > 0.05) (Table 1).

**TABLE 1:** Comparison of the general information of the two groups.

Variables	Control (n = 28)	Experimental (n = 28)	*T/F values*	*P values*
Age (y)	10.56 ± 2.07	11.12 ± 3.84	0.685	0.097
Gender (M/F, n) ^※^	19/9	20/8	0.084	0.771
Height (cm)	140.31 ± 14.77	143.77 ± 18.35	0.776	0.441
Weight (kg)	36.86 ± 15.83	42.38 ± 17.33	1.244	0.219
Lower limb length (cm)	71.93 ± 10.05	73.18 ± 10.30	0.460	0.648

※ “Gender”: the chi-square test.

### 3.2 Temporal and spatial gait parameters

The step width parameter was normally distributed and was therefore expressed as mean ± standard deviation (x ± s). An independent samples *t*-test was used for between-group comparison. In contrast, the remaining data did not conform to a normal distribution and are expressed as median. Accordingly, a Mann–Whitney U test was employed for between-group comparison.

The results demonstrated that walking speed, stride length, step length and single support time in the experimental group were significantly lower than those in the control group (P < 0.05). Conversely, cadence, stride time, step time and double support time were significantly higher than those in the control group (P < 0.05). Notably, there was no significant difference in step width between the two groups (P = 0.086) (Table 2).

**TABLE 2:** Comparison of the temporal and spatial gait parameters of the two groups.

Variables	Control (n = 28)	Experimental (n = 28)	*Z/t values*	*P values*
Cadence (steps/min)	81.50 (62.93,104.25)	113.96 (101.49,126.52)	4.515	<0.001
Walking Speed (m/s)	1.04 (0.81,1.17)	0.60 (0.32,1.01)	−3.881	<0.001
Step Width(m)	0.24 ± 0.07	0.27 ± 0.06	1.749	0.086
Stride Length(m)	1.14 (0.89,1.19)	0.84 (0.58,1.16)	−2.351	0.019
Stride Time(s)	1.06 (0.97,1.19)	1.58 (1.16,1.96)	−4.689	<0.001
Step Length(m)	0.58 (0.49,0.60)	0.35 (0.20,0.57)	−2.616	0.016
Step Time(s)	0.53 (0.50,0.59)	0.81 (0.60,1.00)	−4.371	<0.001
Single Support(s)	0.48 (0.41,0.55)	0.42 (0.37,0.48)	1.994	0.046
Double Support(s)	0.22 (0.20,0.27)	0.40 (0.25,0.81)	−4.631	<0.001

### 3.3 Dynamic balance parameters

The data for the COM and the COP in the coronal (x) and sagittal (y) planes for the two groups did not conform to a normal distribution; therefore, they were expressed as medians and compared between groups using the Mann–Whitney U test.

The results demonstrated that peak COP displacements in both planes and peak COM displacements in the coronal plane were significantly higher in the experimental group than in the control group (P < 0.05). Conversely, the peak COM displacements in the sagittal plane were significantly lower (P = 0.032) (Table 3).

**TABLE 3:** Comparison of the dynamic balance parameters of the two groups.

Variables	Control (n = 28)	Experimental (n = 28)	*Z values*	*P values*
COM-x (mm)	132.20 (97.69,290.32)	225.26 (177.32,321.83)	2.606	0.009
COM-y (mm)	3,623.68 (3,434.86,3822.48)	3,236.09 (3,055.59,3657.03)	−2.147	0.032
COP-x (mm)	1,625.73 (1,318.69,5023.82)	2,140.83 (1797.74,4639.56)	2.212	0.027
COP-y (mm)	5,784.29 (4,621.71,12659.32)	7,534.35 (6,098.21,13479.35)	2.081	0.037

x represents left-right direction; y represents front-back direction.

### 3.4 Joint angle changes

#### 3.4.1 Coronal plane

**FIGURE 4:**
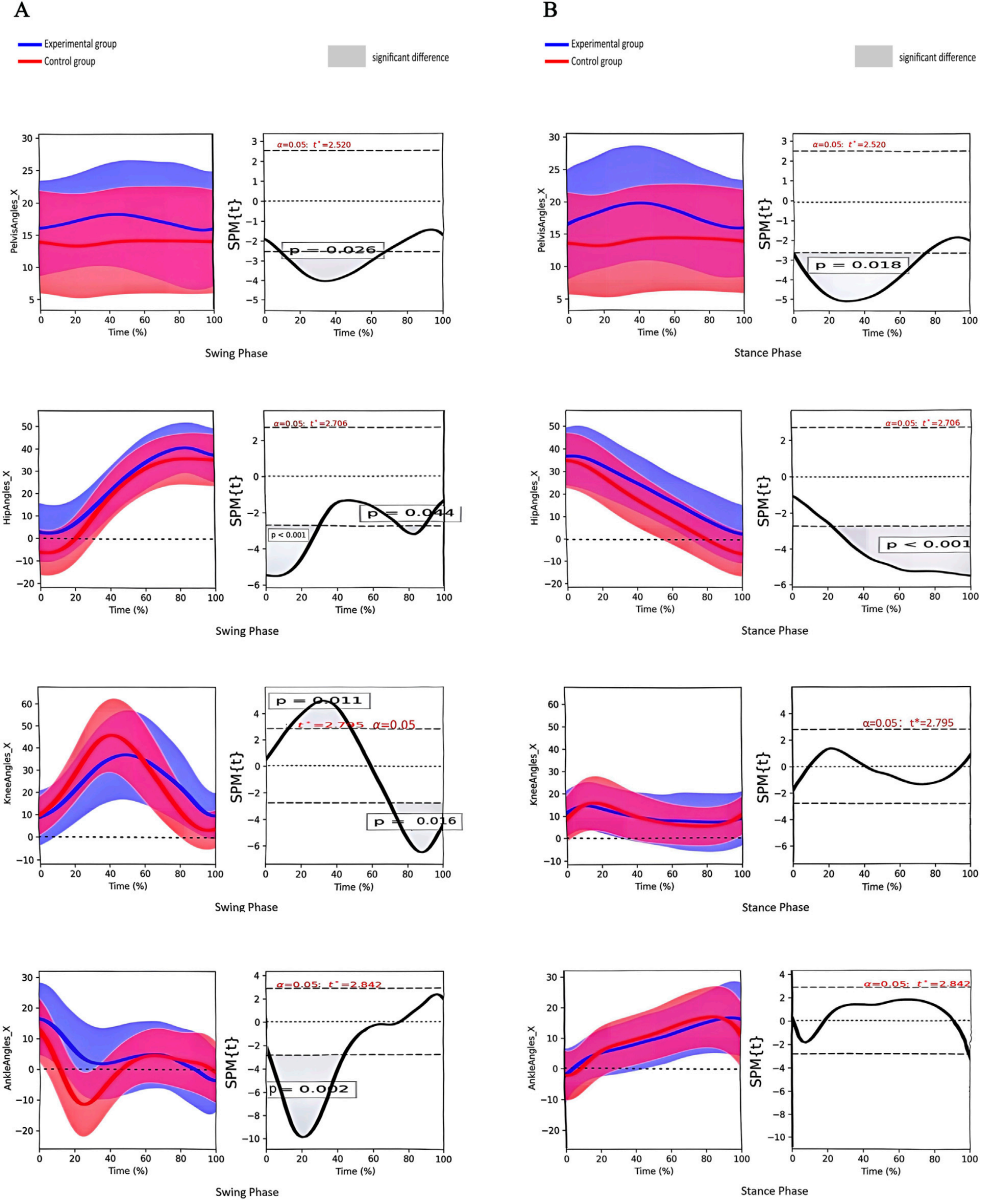
**(A)** Angle change curves of different joints in the coronal plane and paired t-test results of the two groups during the swing phase. Note:①From top to bottom: pelvis, hip, knee and ankle joints; ②The left column shows the angular change curve of each joint in 100% swing phase; ③The right column shows the result of paired t-test between the two groups, and the shaded part represents the P < 0.05 interval. **(B)** Angle change curves of different joints in the coronal plane and paired t-test results of the two groups during the stance phase. Note:① From top to bottom: pelvis, hip, knee and ankle joints; ② The left column shows the angular change curve of each joint in 100% stance phase; ③ The right column shows the result of paired t-test between the two groups, and the shaded part represents the P < 0.05 interval.

There was a significant difference in the angle of pelvic lateral tilt between the two groups during the stance phase and the swing phase. A significant difference was also observed in the hip joint angle during the stance phase and the swing phase. The knee joint angle differed significantly during the swing phase, as did the ankle joint angle (Figures 4A,B).

#### 3.4.2 Sagittal plane

**FIGURE 5:**
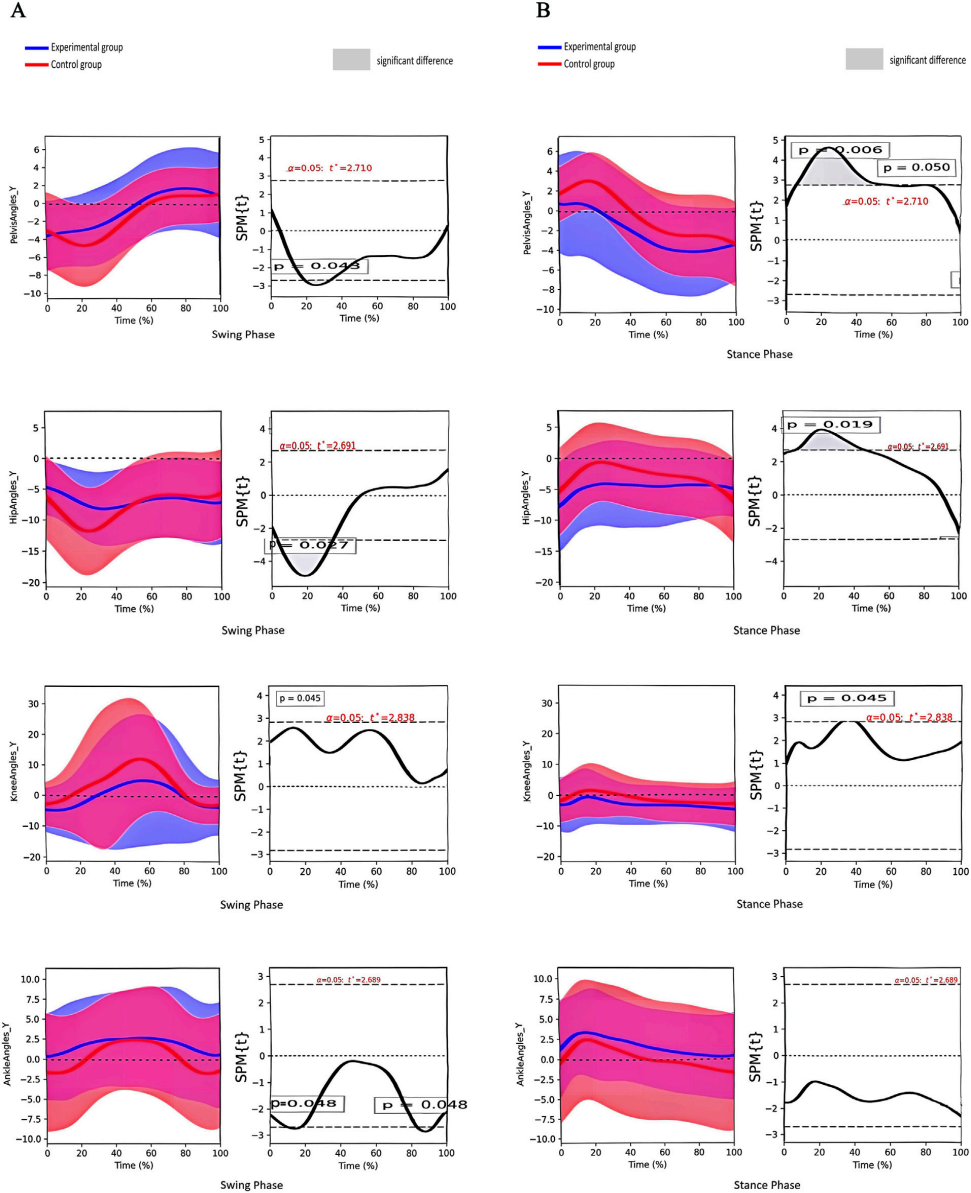
**(A)** Angle change curves of different joints in the sagittal plane and paired t-test results of the two groups during the swing phase. Note:① From top to bottom: pelvis, hip, knee and ankle joints; ② The left column shows the angular change curve of each joint in 100% swing phase; ③ The right column shows the result of paired t-test between the two groups, and the shaded part represents the P < 0.05 interval. **(B)** Angle change curves of different joints in the sagittal plane and paired t-test results of the two groups during the stance phase. Note:① From top to bottom: pelvis, hip, knee and ankle joints; ② The left column shows the angular change curve of each joint in 100% stance phase; ③ The right column shows the result of paired t-test between the two groups, and the shaded part represents the P < 0.05 interval.

There was a significant difference in the anterior/posterior pelvic tilt angle between the two groups during the stance phase and the swing phase. A significant difference was observed in the hip joint angle during the stance phase and the swing phase. The knee joint angle differed significantly during the stance phase. The ankle joint angle varied significantly during the swing phase (Figures 5A,B).

#### 3.4.3 Horizontal plane

**FIGURE 6:**
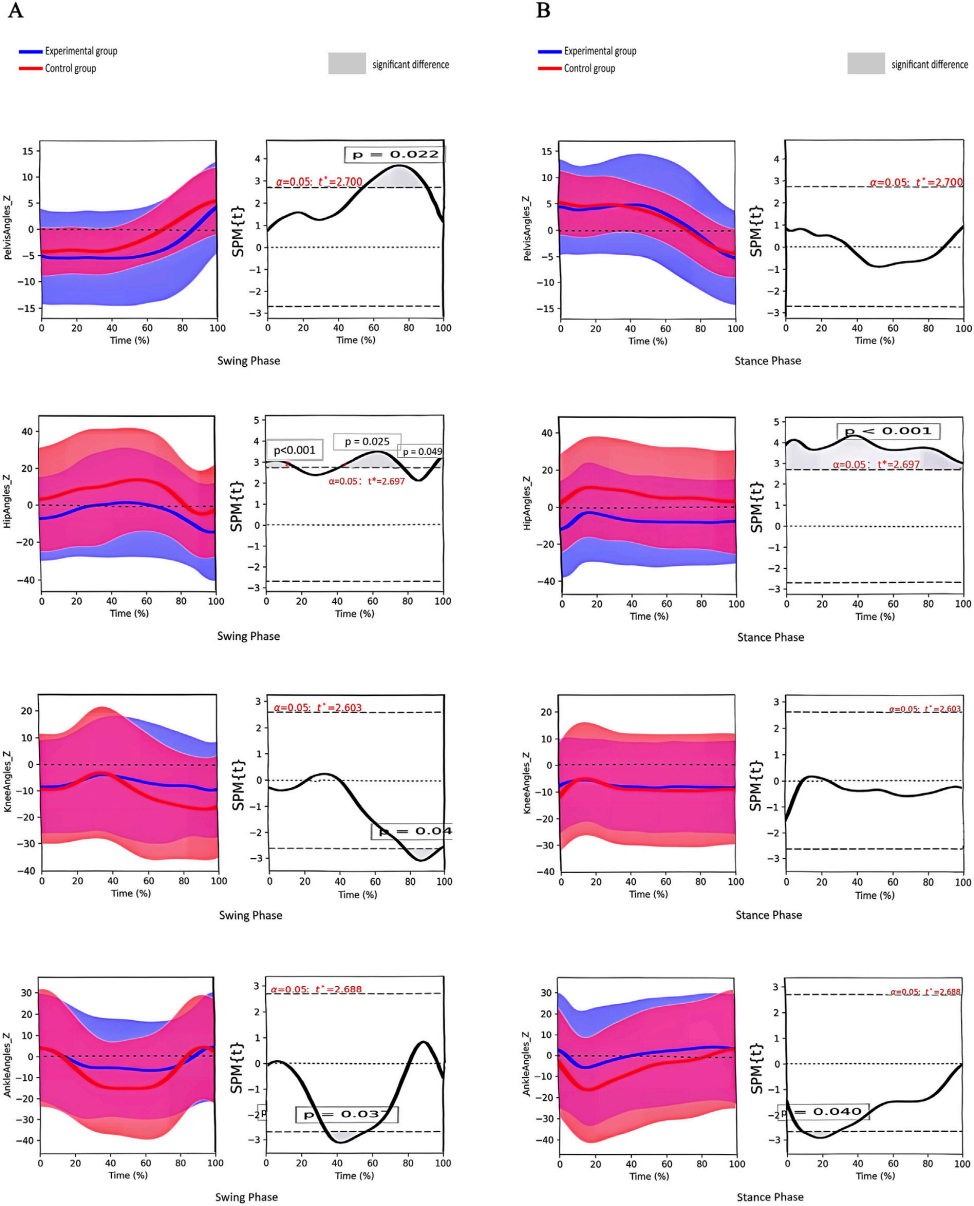
**(A)** Angle change curves of different joints in the horizontal plane and paired t-test results of the two groups during the swing phase. Note:① From top to bottom: pelvis, hip, knee and ankle joints; ② The left column shows the angular change curve of each joint in 100% swing phase; ③ The right column shows the result of paired t-test between the two groups, and the shaded part represents the P < 0.05 interval. **(B)** Angle change curves of different joints in the horizontal plane and paired t-test results of the two groups during the stance phase. Note:① From top to bottom: pelvis, hip, knee and ankle joints; ② The left column shows the angular change curve of each joint in 100% stance phase; ③ The right column shows the result of paired t-test between the two groups, and the shaded part represents the P < 0.05 interval.

There was a significant difference in the horizontal rotation angle of the pelvis between the two groups during the swing phase. A significant difference was also observed in the hip joint angle during the stance phase and the swing phase. The knee joint angle differed significantly during the swing phase. The ankle joint angle varied significantly between the stance phase and the swing phase (Figures 6A,B).

## 4 Discussion

In accordance with the principles of human motor development, during the initial stages of independent walking, children’s lower limb hip joints frequently adopt an abducted and externally rotated position with a relatively large support surface and insufficient hip and knee joint extension (Willerslev-Olsen et al., 2015). As children grow older, they refine their mature gait patterns through exercise, gradually improving walking stability. During walking, running and walking up and down stairs, the COP alternates between the feet in accordance with their alternating positions, and the COM drives the body through space by constantly exceeding the plantar support area (Õunpuu et al., 2015).

Our study demonstrated that the peak displacements of the COM and the COP in the coronal plane were greater for children in the experimental group than for those in the control group. This indicates that the body’s centre of gravity and plantar pressure of children with SCP swung more laterally than those of typically developing children during walking. These findings are consistent with the results of the present study, which showed that the angle of the hip joint in the coronal plane of the test group differed considerably from that of typically developing children from the mid-support phase (mid-stance) to the early swing phase (initial swing) (11%–65% of the gait cycle). Mid-stance is a component of the unipedal support phase, during which the hip on the support side transitions from a neutral to an extended position. This is done to maintain the trunk in an upright position and to counteract the gravitational loads on the lower extremity on the swing side. In the general population, the moment of gravity is counteracted by a low level of hip abduction moment and a passive acceleration moment. In contrast, children with SCP require a passive increase in hip abduction moment through compensation by lateral pelvic tilt on the support side due to inadequate control of the pelvis and trunk and on the swing side through pelvic rotation (a considerable difference in the gait cycle from 77% to 95% in the horizontal plane) and internal rotation of the hip joint. The rotation of the hip joint (considerable difference in the gait cycle from 73% to 89%) enables the lower limb to be thrown forwards to complete the stride, which results in increased lateral movement of the COM and the COP on the support side (Tracy et al., 2019; Rethwilm et al., 2021). It can be observed that the greater the range of movement of the COM and the COP, the higher the mean velocity, which consequently increases the risk of loss of balance and fall to the side.

Differences in the COM in the sagittal plane between the two groups suggest that children with SCP lag considerably behind typically developing children in anterior drive during walking, associated with ankle stirrup weakness (Özden et al., 2023; Yun et al., 2023). The study revealed that ankle dorsiflexion angles were greater in children with SCP than in typically developing children during the pre-swing period (55%–59% of the gait cycle), suggesting insufficient propulsive force during toe-off. In normal gait, the ankle joint generates forward propulsion through plantarflexion. However, the abnormal increase in ankle dorsiflexion and valgus angles due to a crouching posture in children with SCP limits forward COM movement, reducing stride length (Lerner et al., 2016). Stride length is an important factor influencing the sagittal plane trajectory of the COM (Valenciano et al., 2022; Peia et al., 2023). The reduction in forward COM displacement indicates a shorter stride length for the child, which also aligns with the findings of the temporospatial gait parameters in the study.

Some scholars have proposed that controlling foot spatial position during the swing phase represents the optimal strategy for reorienting or altering COM movements (Rapson et al., 2023). However, children with SCP often exhibit persistent motor and postural deficits such as crouching, hopping and stiff-knee gaits, leading to postural instability and rigidity. This movement strategy results in poor separation and coordination during walking and a tendency for the body to move as a bloc during ambulation. Children with SCP are unable to effectively control the lower limb on the swing side to drive forwards accurately and smoothly (Kawaguchi et al., 2024).

Sutherland et al. observed that children’s gait matures with changes in five key parameters, including increased single support phase time, walking speed and stride length, as well as decreased cadence and ankle spacing relative to pelvic width (Sutherland et al., 1980). This study found that the walking speed, stride length and single support time of children with SCP were lower than those of the control group. This is consistent with the characteristics of delayed gait development in children with SCP (Gibson and Stork, 2021). The increase in cadence and double support time indicates that children with SCP reduce single support time by accelerating their cadence to maintain body stability during the dynamic imbalance period of body weight transition, thereby avoiding falls (Bach et al., 2021). Prior research has indicated that children with SCP exhibit longer anterior-posterior COP sway paths while standing and, consequently, greater COP displacements during ambulation than typically developing children of similar age (Szopa and Domagalska-Szopa, 2024), explaining the greater peak COP displacement in the sagittal plane observed in this study.

The findings of the study indicate that children with SCP exhibit differences from their typically developing peers in terms of dynamic balance, temporal-spatial parameters and kinematic parameters. Consequently, PT interventions may be considered in a clinical setting to address these discrepancies and thereby enhance walking ability.

This study revealed the main kinematic abnormalities during the support and swing phases in children with SCP, including limited hip mobility, insufficient ankle propulsion and poor control of the centre of gravity. Robotic-assisted gait training (RAGT) offers a potential intervention for addressing these functional deficiencies.

In recent years, exoskeleton robots – an important tool in the field of PT – have been widely used in the rehabilitation training of children with CP. A substantial body of evidence suggests that these devices can effectively enhance the gait pattern of children with SCP, with the degree of improvement dependent on the duration of intervention (Hunt et al., 2022). To minimise additional energy expenditure for children, an exoskeleton robot weighing no more than 2.5 kg and capable of delivering high torque is recommended. This type of robot can be used for RAGT, typically consisting of sessions lasting 9–18 min, 3 times per week, for a minimum of 8 weeks. This approach has been shown to improve the characteristic gait pattern of SCP (Rossi et al., 2013; Orekhov et al., 2020; Corsi et al., 2021). Robotic-assisted gait training not only substantially increases stride length but also improves walking speed and optimises abnormal spatiotemporal gait parameters (Betancourt et al., 2019). Additionally, a knee exoskeleton has been shown to help children with SCP enhance hip extension during the support phase, enabling a more upright trunk posture and correcting the crouched gait (Lerner et al., 2017; Chen et al., 2021), whereas unrestrained ankle exoskeletons and P. REX considerably increase peak ankle plantarflexion and the toe-off moment (Chen et al., 2021; Lerner et al., 2019).

Notably, these findings provide compelling clinical evidence for the improvement of lower extremity kinematic and kinetic parameters during ambulation in children with SCP. This refined approach not only highlights the potential of RAGT to improve the quality of life for children with SCP but also underscores its importance as a therapeutic tool in paediatric rehabilitation. Future research should continue to explore the optimal parameters for RAGT to maximise its benefits for patients.

It should be noted that this study is not without limitations. First, the sample size was relatively small, which may have introduced some errors in the findings. Second, the study population comprised solely children with SCP and typically developing children aged 6–12 years. No studies on gait variability at other ages, particularly below the age of 6 years (a period of immature gait development), were conducted (Carollo et al., 2018). Third, this study concentrated solely on the discrepancies in the kinematics of the pelvis and lower limbs, with no investigation of the head, neck, trunk and upper limbs. Fourth, this study did not address the characterisation of changes in key muscle strength aspects of walking. Finally, based on the consideration of methodological consistency, a one-sided analysis strategy was adopted in order to avoid the statistical bias that may be introduced by the mixing of bilateral lower extremity data. Therefore, in the study of both the spastic diplegic group and the normal child group, the same side (left) was selected as the main object of analysis, so as to more sensitively capture the dynamic differences during gait between the two groups. Future studies could include bilateral data and statistically compare the effects of different limb choices on dynamic equilibrium parameters to further validate the robustness of the current conclusions.

The findings of this study suggest that well-designed large-sample multicentre clinical studies can be conducted in the future to gain a deeper understanding of the differences in whole-body kinematic parameters between same-age children with SCP and children without the condition. Furthermore, incorporating a comparison of electromyographic signals and other indices could further enrich the results regarding the variability of differences between the dynamic balance function and the kinematics and dynamics of walking in children with SCP and same-age typically developing children.

## 5 Conclusion

The lateral dynamic balance control ability of children with SCP is found to be inferior to that of typically developing children of the same age when walking, which consequently increases the risk of lateral falls. Children with SCP are observed to be limited by toe-stomping power, and their forward driving force is insufficient during walking, resulting in walking speed and stride length lagging behind those of typically developing children. The combined application of RAGT, FES and WBV with PT, along with a well-designed exercise programme, has been demonstrated to enhance the dynamic balance abilities of children with SCP, improve their walking stability and rectify abnormal gait patterns.

## Data Availability

The original contributions presented in the study are included in the article/supplementary material, further inquiries can be directed to the corresponding author.
